# Type 1 Innate Lymphoid Cell Biology: Lessons Learnt from Natural Killer Cells

**DOI:** 10.3389/fimmu.2016.00426

**Published:** 2016-10-12

**Authors:** Yuhao Jiao, Nicholas D. Huntington, Gabrielle T. Belz, Cyril Seillet

**Affiliations:** ^1^Molecular Immunology Division, Walter and Eliza Hall Institute of Medical Research, Melbourne, VIC, Australia; ^2^Department of Medical Biology, University of Melbourne, Melbourne, VIC, Australia; ^3^School of Medicine, Tsinghua University, Beijing, China

**Keywords:** innate lymphoid cells, immunity, immune protection, lymphocyte subsets, GVHD, tumor rejection

## Abstract

Group 1 innate lymphoid cells (ILCs) comprise the natural killer (NK) cells and ILC1s that reside within peripheral tissues. Several different ILC1 subsets have recently been characterized; however, no unique markers have been identified that uniquely define these subsets. Whether ILC1s and NK cells are in fact distinct lineages, or alternately exhibit transitional molecular programs that allow them to adapt to different tissue niches remains an open question. NK cells are the prototypic member of the Group 1 ILCs and have been historically assigned the functions of what now appears to be a multi-subset family that are distributed throughout the body. This raises the question of whether each of these populations mediate distinct functions during infection and tumor immunosurveillance. Here, we review the diversity of the Group 1 ILC subsets in their transcriptional regulation, localization, mobility, and receptor expression, and highlight the challenges in unraveling the individual functions of these different populations of cells.

## Introduction

Innate lymphoid cells (ILCs) are members of an expanding family of immune cells that provide the first line of defense against invading pathogens and they also contribute to tissue repair (1). Unlike T and B lymphocytes, ILCs develop independent of the recombinant activating gene (RAG). They have been classified into three main groups (groups 1–3) based on their cytokine and transcription factor expression that aligns them with the subsets used to categorize different CD4^+^ T helper (Th) cell populations (2). The ILC1 family, such as Th1 cells, is composed of the T-bet-expressing cells that secrete interferon (IFN)-γ. Group 2 ILCs (ILC2s), initially described as “natural helper cells” (3), or nuocytes (4), express GATA-3 similar to Th2 CD4^+^ T cells, while ILC3s produce IL-17 and/or IL-22 and express the transcription factor RORγt (5). This latter subset is proposed to be equivalent to Th17 and Th22 cells.

Innate lymphoid cells have been the focus of extensive investigation during the last 5 years, in part due to their potential impact on human health and disease. Several studies have uncovered significant heterogeneity and plasticity within the ILC subsets, a feature which highlights that the field may have overlooked delineating the individual contributions of different ILC subsets and identifying their contributions to immunity. This is especially important in the Group 1 ILCs that were originally thought to represent a single homogeneous population composed of natural killer (NK) cells alone. It has now been found that this group is heterogeneous and includes a second subset of T-bet-dependent IFN-γ-producing innate cells that localize principally in tissues (6). Given this diversity within the group 1 ILCs we will refer to the respective subsets as NK cells and ILC1s. The finding that two IFN-γ-producing subsets exist also raises the possibility that previous analyses of NK cells may have overlooked the contributions of ILC1s as they express similar surface markers, including NKp46, CD122, and NK1.1, to NK cells. However, it is now recognized that NK cells and ILC1s are likely to have quite distinct roles in protective immunity as they differ in multiple aspects, including their transcriptional regulation, localization, mobility, and receptor expression. The challenge in teasing apart the individual functions of these different populations of cells relies largely on the identification of unique markers to characterize each of the populations. In this review, we provide an overview of the different Group 1 ILC populations and highlight the current understanding of their roles in immune protection.

## Heterogeneity and Plasticity of the Group 1 ILCs

Group 1 ILCs were initially thought to include only the prototypic member, the NK cell. Even early on though, heterogeneity had already been described among NK cells found in different tissues, such as the liver, the thymus, and the uterus. These NK cells differentially expressed a number of surface molecules, suggesting that much greater diversity existed (7). Classically, NK cells have been defined as CD3^−^NK1.1^+^ with NKp46 expression being identified somewhat later as a more specific marker for mature NK cell cells that are found in secondary lymphoid tissues, such as lymph nodes and the spleen (8). A further subset of ILC1s, initially thought to be NK cells, was found in the liver and expressed the molecule tumor necrosis factor-related apoptosis-inducing ligand (TRAIL) (9). More recently, it has been shown that the intrahepatic ILCs comprise a distinct lineage of ILC1s which develops separately from NK cells (10). ILC1s have also been found in the thymus and express high levels of the IL-7 receptor α (IL-7Rα, CD127) and Gata-3 (11). A further distinction has been elucidated between NK cells and ILC1 as the latter are generally localized within tissues. This has led to their designation as “tissue-resident” cells. This contrasts with the majority of NK cells that at steady-state circulate continuously patrolling for infected or malignant cells, while ILC1 rarely recirculate and appear to be generated by progenitor cells in the tissues or self-renew locally (6, 12). How the behavior of these two subsets of cells might be altered during inflammation is not yet clear. It is possible that in response to infection bone marrow-derived precursors become key contributors to the pool of tissue-resident ILC1s to meet the significantly increased demands of responding to a pathogen infection. Some evidence for such a model is provided by the adoptive transfer of bone marrow ILC progenitors into sublethally irradiated mice that were then able to generate liver and intestinal ILC1s (13, 14).

### Conventional NK Cells

Natural killer cells represent 5–15% of circulating lymphocytes in humans and 2–5% in mice. It is well established that tumor growth is in part influenced by the activity of the immune system. However, it has only recently been appreciated that tumor cell avoidance of immune detection also contributes to cancer development in humans. NK cells were identified in the early 1970s due to their ability to spontaneously kill leukemia cells and they have now been strongly implicated as key effectors in cancer immunosurveillance, transplantation rejection, and early viral immunity (15).

The cytokine IL-15 is essential for most facets of NK cell biology. It binds to IL-15Rβ/γ_c_ heterodimers on the surface of NK cells to drive their survival and proliferation. It also primes NK cell activation resulting in the production of pro-inflammatory cytokines and lytic granules. IL-15 binding results in activation of the JAK1/3 and STAT5 signaling pathways and this induces STAT5-target genes, such as the pro-survival gene *Myeloid cell leukemia 1* (*Mcl1*) and the negative regulator of IL-15 signaling (16), cytokine-inducible SH2-containing protein encoded by *Cish*, which are essential for NK cell survival and homeostasis (17). The NK cells also express multiple activating and inhibitory receptors of the NKG2 and Ly49 (KIR in humans) family (18). The activation of the NK cells relies on the dynamic balance between activating and inhibitory pathways allowing the cells to rapidly sense changes in the environment. NK cells can kill infected or transformed cells through cytolytic mechanisms (granzymes or perforin) or engagement of death receptors.

### ILC1s

**Figure 1:**
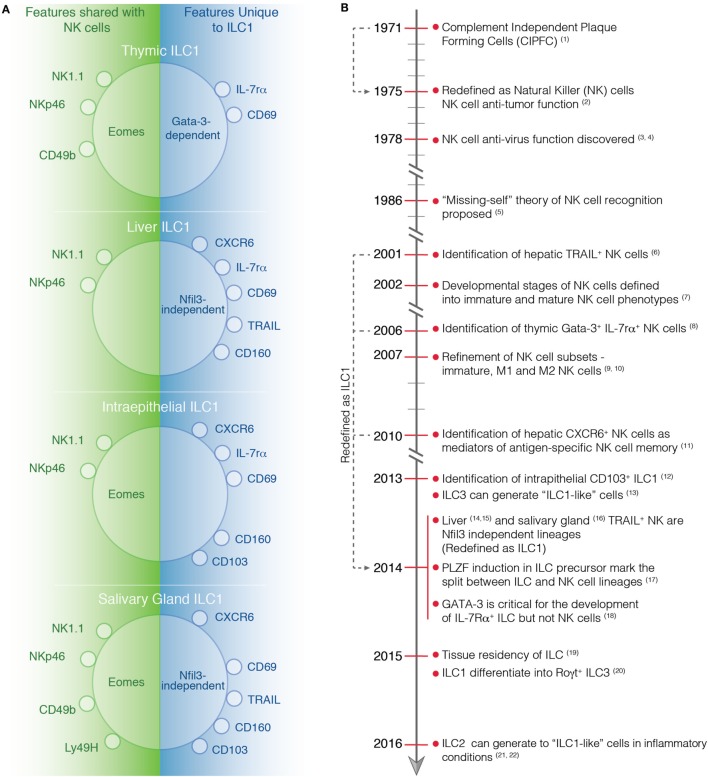
**(A)** Phenotype of tissue-specific ILC1s and NK cells at steady-state. Characteristic markers of ILC1 in the thymus, liver, intestine, and salivary gland are depicted. On the left, the markers that are expressed by both ILC1s and NK cells are shown while on the right the marker that are only expressed by ILC1s are indicated. **(B)** Timeline highlighting important discoveries in the identification and characterization of NK cells and ILC1s. (1) Thornthwaite et al. (21). (2) Kiessling et al. (22). (3) Anderson (23). (4) Santoli et al. (24). (5) Karre et al. (25). (6) Takeda et al. (26). (7) Kim et al. (27). (8) Vosshenrich et al. (11). (9) Hayakawa et al. (28). (10) Huntington et al. (29). (11) Paust et al. (30). (12) Fuchs et al. (31). (13) Bernink et al. (32). (14) Seillet et al. (10). (15) Daussy et al. (33). (16) Cortez et al. (34). (17) Constantinides et al. (13). (18) Yagi et al. (35). (19) Gasteiger et al. (6). (20) Bernink et al. (36). (21) Bal et al. (37). (22) Ohne et al. (38).

ILC1s are found at steady state in virtually all tissues, but are enriched in the liver, uterus, skin, salivary glands, and the gut (19). In contrast to NK cells that express integrin α2 (CD49b), ILC1s express high levels of integrin α1 (CD49a) but lack integrin α2 and the T-box transcription factor eomesodermin (Eomes). Conventionally, Eomes expression has been thought to be restricted to NK cells alone and not expressed by ILC1s. However, this does not appear to be a universal rule as ILC1s in some tissues can also express Eomes highlighting that the use of transcription factor expression to demarcate subsets is not necessarily definitive in all settings (Table 1; Figure 1). Similarly, while CD49a expression is restricted to ILC1 at steady state, following activation, NK cells can also express this marker (20). This incomplete clarity in defining the subsets highlights their complexity and the requirement to dig deeper into understanding the distinct roles of ILC1s and NK cells *in vivo*.

**Table 1:** **Phenotype of NK cells and tissue-specific ILC1**.

	NK1.1	NKp46	CD49a	CD49b	CXCR6	CD127	TRAIL	CD160	CD226	Ly49E	CD11b	KLRG1	CD62L	EOMES
NK cells	+	+	–	+	–	–	–	–	+	–	+	+	+	+
Liver ILC1	+	+	+	–	+	±	+	+	++	+	–	–	–	–
Thymic ILC1	+	+	n.d.	+	n.d.	+	n.d.	n.d.	n.d.	n.d.	–	–	n.d.	+
IEILC1	+	+	+	–	+	+	+	+	+	–	–	–	–	–
Salivary ILC1	+	+	+	+	+	–	+	+	n.d.	n.d.	–	–	–	+

*±, heterogeneous expression; n.d., not determined*.

#### Liver ILC1s

Natural killer cells that differed from conventional NK cells were identified in the liver by the Smyth group who described a unique subset of NK1.1^+^ cells that expressed TRAIL (9). Initially, they were considered to be immature NK cells as they were enriched for CD27^+^CD11b^−^ cells that lacked expression of most of the Ly49 molecules and CD49b (9). It is now clear that this population is a distinct ILC1 lineage that in contrast to NK cells, which does not require the transcription factor nuclear factor interleukin 3 (Nfil3) for development (10) and is tissue-resident being located almost solely in the liver at steady state (12) (Figure 1B). Interestingly, liver ILC1s share with other tissue-resident lymphocytes the dependency on the transcription factor Hobit, while ILC1s in other tissues do not (39). Transcriptomic analysis revealed that liver ILC1 gene expression was quite distinct from NK cells (10). ILC1s display a unique pattern of chemokine receptors and adhesion molecules distinct from NK cells. These are likely to be important in maintaining their resident positioning in tissues and include the receptors CXCR6, CXCR3, CD103, CD49a, CD69, and CD39 (10, 12, 33). By contrast, NK cells express CX3CR1, CD62L, S1PR1, and S1PR5 that are not found in the ILC1. Liver ILC1s also express their own pattern of cytokine receptors (IL-7R, IL-17RD, IL-21R, and TGF-βR) together with a set of regulatory molecules, such as CD200R, PD1-L, ICOSL, and Lag3 (10, 33).

It is now clear that intrahepatic ILC1s are resident cells; however, how the stability of this population maintained is not clear. Several studies have been undertaken to understand this feature of ILCs but the outcomes have not been consistent (12, 40). One possibility for these discrepancies could be that mouse strains of different backgrounds that have been used as recipients of adoptively transferred ILC1s may impact the development and expansion of these cells. For example, liver ILC1s transferred into sublethally irradiated C57BL/6 mice maintained their original phenotype (12), while ILC1s transferred into immunodeficient Rag2^−/−^γ_c_^−/−^ mice differentiated into NK cells that then expressed Eomes and lost surface TRAIL expression (40).

Intrahepatic ILC1s have also been identified in man (41). Interestingly, these cells share many phenotypic traits with mouse liver ILC1s, including the expression of CD49a, NKp46, DNAM-1, and T-bet, but lack Eomes (41). They appear to also share similar functional capacity as they produce high levels of IFN-γ, TNF-α, and GM-CSF, a poor degranulation response and low levels of cytotoxic effector molecules. Despite the conservation of characteristics between man and mouse, the actual role of the ILC1s in the liver is currently unknown. However, it has been shown that these cells do have a unique capacity to generate immune memory responses against hapten and viral antigens (30). Unexpectedly, liver ILC1s could mediate recall responses in an antigen-specific manner. Thus far, however, neither the mechanisms nor the receptors involved in the specific recognition of the antigens are known and will require further investigation.

#### Thymic ILC1s

NKp46-expressing cells that reside in the thymus were first described as NK cells (11). However, a more recent understanding of their phenotype suggests that they fit more closely with the description for ILC1. Thymic ILC1s differ from conventional NK cells as they express IL-7Rα and require the transcription factor Gata-3 for development (11) similar to other ILC1s (35) and should, therefore, be considered as members of the ILC1 subset (Figure 1B). Bone marrow-derived NK cells do not depend on Gata-3 during differentiation although it is important for them to produce IFN-γ during maturation. Thymic ILC1s display a phenotype that is intermediate between classical NK cells and ILC1s again adding to the debate around the classification of these cells. Similar to other ILC1s, thymic ILCs express low levels of Ly49 and high levels of IL-7Rα, CD11b, and CD69. They also express Eomes, a transcription factor normally characteristic of NK cells. Thymic ILC1s can develop *in vitro* and *in vivo* from the CD4^−^CD8^−^ (DN1) subset of immature thymocytes, indicating that they do not arise from committed precursors in the bone marrow (42). To date, relatively little is known about the origins and contributions of thymic ILC1s to immune protection or homeostasis. Further work will be required to characterize these cells and establish their role in the thymus.

#### Intestinal ILC1s

At least three phenotypically distinct subsets of ILC1s can be found in the gut. In the lamina propria (LP) both NK cells and ILC1s (LP ILC1) have been described, while a third subset of ILC1s reside in the epithelium (ieILC1). LP ILC1s are characterized by high expression of IL-7Rα and the lack of Eomes while NK cells lack IL-7Rα and express Eomes (14). In humans, a subset of CD127^+^ IFN-γ-producing ILC1s have been identified in the gut and have been shown to be enriched in the intestine of Crohn’s disease patients (32). They are also enriched for CD69, but like ILC1s produce high level of IFN-γ and lack of Eomes expression. These cells do not express NKp46 or CD56 but express CD161, a marker commonly expressed by the ILC3 and ILC2 subsets.

An additional ILC1 subset has been reported in the gastrointestinal epithelia in both human and mice (31). These intraepithelial ILC1s (ieILC1s) are characterized by the expression of lymphocyte markers, such as CD103 and CD160 (31). While human ieILC1s express NKp46, CD56, and NKp44, they also express Eomes similar to human LP ILC1s in contrast to murine ILC1 that do not express Eomes.

#### Salivary Gland ILC1s

ILC1s are found in the salivary gland and like those in some other sites express TRAIL, CD49a, CD103, and CD69 and express Eomes (34) (Table 1, Figure 1). Interestingly they develop independent of the transcription factor Nfil3 that is similar to liver ILC1s, suggesting that salivary ILC1s may be distinct from NK cells that depend on Nfil3 for development (10). Recently, TGF-β signaling has been implicated in the maintenance of salivary ILC1s (43). The lack of TGF-β signaling was associated with reduced numbers of ILC1 and impaired expression of CD49a, CD103, and CD69 in the salivary glands. By contrast, TGF-βR2 deficiency had minimal impact on the phenotype of ILC1s found in the gut and the liver, suggesting that other factors guide their differentiation (43).

## ILC1 Plasticity

Tools to investigate the distinct roles of ILC1s and NK cells are currently limited. At steady state, ILC1s and NK cells are distinguished based on the expression of surface markers (e.g., TRAIL, CD49a, CXCR6) and transcription factors (e.g., Eomes, PLZF). However, inflammation can modify these features complicating our ability to track *bona fide* ILC1s and NK cells. Surface markers, such as TRAIL or CD49a can be upregulated during MCMV infection or following exposure to cytokines, such as IL-2, IFN-γ, or IL-15 (20, 44, 45). During inflammation, cytokines can also divert ILC identity as has been shown for ILC2s and ILC3s, which can acquire a phenotype consistent with ILC1s. These so called “ILC1-like” cells that produce IFN-γ can arise from Rorγt^+^ NKp46-expressing ILC3s in the intestine following stimulation with cytokines, such as IL-12 and IL-18 (32, 46). LP ILC1s are also influenced by the microenvironment differentiating into Rorγt^+^ ILC3s when exposed to IL-23 and IL-1β. This process was enhanced in the presence of retinoic acid while IL-12 could reverse the transition (36). Similarly in the lung, IL-1β and IL-12 can induce the conversion of ILC2s to ILC1-like cells (37, 38). These findings highlight that ILC are capable of rapid adaption to changes in environmental cues induced by pathogens or inflammation.

## Contribution of the Group 1 ILC to Pathogen Responses

Early resistance against intracellular bacteria has been attributed to the activation of phagocytic cells by NK cells through their capacity to produce IFN-γ (47, 48). However, the beneficial role of NK cells in these infections became contentious when more recent studies showed that depletion of NK1.1^+^ cells led to a reduction of bacterial load, a result that was not be concordant with the proposed positive role for NK cells. It would appear that during infection with *Listeria monocytogenes*, excessive IFN-γ produced by NK cells *via* activation through the costimulatory molecule CD27 impairs innate anti-bacterial defenses (49). This effect was not limited to Listeria infection (49, 50) as NK cell depletion also protected against lethal *Escherichia coli* (51) and *Streptococcus pneumonia* infection (52). An additional explanation for these paradoxical results, however, is that ILC1s and NK cells make differential contributions to defense against infection. These studies were conducted before the discovery of the ILC1s and have inevitably categorized all cells expressing NKp46 and NK1.1 and secrete IFN-γ and TNF-α as putative “NK cells.”

Re-investigation of the role of NKp46-expressing cells in the control of parasites, such as *Toxoplasma gondii*, revealed that ILC1s are indeed a major source of IFN-γ and TNF-α following oral infection (14). By contrast, NK cells or NKp46^+^ ILC3s appeared to have only minor contribution in these settings. However, whether ILC1s are solely responsible for the control of infection remains to be determined (14). In these experiments, the mice carried a germline deletion of T-bet resulting in a deficiency in their entire hematopoietic compartment and loss of IFN-γ expression in ILC1s, NK and NKp46^+^ ILC3s. These experiments, therefore, do not delineate the role of T-bet in the various different immune populations that might control infection. Although adoptive transfer of ILC1 into Rag2^−/−^γ_C_^−/−^ mice was found to reduce the Toxoplasma pathogen load, it only partially restored the recruitment of monocytes to a level similar to those observed in T-bet^−/−^ mice. Similarly, in response to *Clostridium difficile*, ILC1s have been shown to be the major contributor to IFN-γ production during acute infection rather than NK cells (53). Early protection against *C. difficile* was mediated by T-bet-expressing ILC1s in a IFN-γ-dependent manner.

These results collectively imply that the enhanced capacity of ILC1s to produce IFN-γ outstrips that of NK cells during enteric infection such that ILC1s may be the main drivers of immune protection. In addition, the potential plasticity of ILCs allowing the polarization of ILC3s toward an ILC1-like phenotype may further favor efficient IFN-γ production by ILC1s. However, due to the paucity of models that allow targeted deletion of ILC1 alone, it remains complicated to formally demonstrate the specific and non-redundant roles of ILC1. Further characterization of the ILC1 populations and identification of the transcription factors regulating their development may shed light on new approaches for *in vivo* models with which ILC1s functions can be specifically assessed.

## Contribution of the Group 1 ILC in Cancer and Inflammation

### Tumor Rejection

Natural killer cells are able to distinguish transformed cells from normal healthy cells, in part by their reduced expression of major histocompatibility complex class I (MHC-I) molecules, the founding feature of the “missing-self” hypothesis. Both *in vivo* and *in vitro* experiments in humans and mice have demonstrated that MHC-I deficient tumor cells are more sensitive to NK cell-mediated cytolysis (25). However, the mechanisms regulating the activation and inhibition of NK cells are more complex than the original “missing-self” hypothesis (54). Studies have shown that cytotoxicity of NK cells against transformed cells is governed by both cytokines and activating and inhibitory receptors. To suppress tumors, NK cells can cause direct cell target death by granule-mediated lysis, receptor mediated cytotoxicity (Fas/FasL or TRAIL death pathway) (55, 56), or antibody-dependent cell cytotoxicity (ADCC) (57). Besides direct killing of tumor cells, NK cells can secrete TNF-α and IFN-γ to drive the Th1 polarization in the tumor microenvironment to further strengthen adaptive cytotoxic responses to tumor cells (58).

In contrast to NK cell activation, ILC1s have not been reported to exhibit crosstalk with other cells through activating and inhibitory signals. Instead, they are activated by cytokines, such as IL-12, IL-15, and IL-18, which are also able to drive NK cell activation (31). Following activation, ILC1s can also produce TNF-α and IFN-γ. It has been proposed that ILC1s should be classified as innate “helper-like” cells distinguishing them from NK cells that would represent cytotoxic ILCs (14), however, it has been shown that liver ILC1s are also able to recognize and kill target cells albeit perhaps less efficiently than NK cells (30, 59). This killing capacity would enable tissue-resident ILC1s to have potentially important roles in controlling transformed cells at an early development stage prior to their dissemination throughout the body. Recently, it was reported that early stage cell transformation can induce a local tissue-resident lymphocyte response accompanied by expression of Granzyme B and potentially cytolytic activity toward the transformed cells (60). These tumor-associated ILC1s appear to be transcriptionally distinct from, but functionally related to, conventional NK cells and differ from ILC1s found at steady state. These cells, though, were dependent on IL-15 but did not require *Nfil3* for development similar to ILC1s. These data suggest that tumor-associated ILC1s are developmentally distinct from NK cells as it has been shown for liver (10) and salivary gland ILC1s (34). However, it has been also shown that Nfil3 is not required for mature NK cell homeostasis and that *Nfil3*-deficient mice are able to generate and maintain NK cell memory in response to MCMV infection (61).

### Transplantation and Graft-versus-Host Disease

A common clinical situation where both acute and chronic inflammation occurs is transplantation. Either solid organ or hematopoietic cell transplantation (HCT), including bone marrow transplantation (BMT), can lead to both host and donor immune reactions, mainly attributed to mismatched MHC molecules from the host and the donor. These immune reactions involve inflammation of multiple organs and are categorized into host-versus-graft disease (HVGD) and graft-versus-host disease (GVHD) reflecting the origins of target and activated immune cells. When host immune cells react against donor cells, elevation in serum cytokine levels and multi-organ inflammation gives rise to HVGD and typically graft failure results. Conversely, GVHD is the disease associated with systemic inflammation caused by activation of donor immune cells that damage the host tissue, a very common clinical complication after HCT. The alloreactivity from donor NK cells against recipient cells can cause selective cytolysis and damage to host cells and tissues, suggesting that it may be entirely deleterious but paradoxically this effect sets the scene for a potent graft-versus-leukemia (GVL) effect that effectively removes leukemic cells leading to positive outcomes. Alloreactive NK cells reduce GVHD by eliminating recipient antigen-presenting cells that drive immune cell activation (62). In a murine model of MHC-matched allogeneic BMT, the infusion of Ly-49 (a member of the inhibitory receptor family) mismatched NK cells contributed significantly to the anti-tumor effect while preventing GVHD (63). The infusion of pre-activated NK cells with a combination of cytokines, such as IL-12/15/18, strongly suppressed acute GVHD (64). These NK cells were found to maintain their expression of Eomes and T-bet *in vivo* which they exhibited prior to transfer which suggests that the key effectors in this setting are NK cells and not ILC1, which have a different profile (64).

Interestingly, mice reconstituted with cells that lack NKp46 expression had greatly exacerbated GVHD and increased mortality due to increased commensal bacteria infection (65). As both NK cells and ILC1s express NKp46, this clinical outcome may reflect the involvement of both cell types but it is not clear how NK cells and ILC1s individually contribute to the disease progression. To date, only a single study has reported a protective role for ILCs in GVHD (66). In this study, patients that did not develop acute GVHD had increased proportions of skin-homing ILC1s, NCR^−^ ILC3s and gut-homing ILC2s, suggesting that ILCs can indeed provide protection against the development of GVHD (66). It was observed that following transplantation, patients that developed more severe GVHD had fewer circulating ILC1s when compared with healthy controls. Mobilization of ILC1s following treatment was associated with elevated expression of the early activation marker CD69, and the tissue homing markers CLA and CCR10 which correlated with less severe progression of GVHD. Nevertheless, the recovery of ILCs following conditioning radio-therapy and chemotherapy was slow compared with other immune cell types and the reconstituting ILCs were derived from the donor. It appears that both ILC1s and NK cells from donors can exert protective roles to restrict the progression of GVHD while simultaneously contributing to the GVL effect. This suggests that balancing the ratios of donor and host ILC1s/NK cells through conditioning prior to BMT may be important for high engraftment and robust GVL effects and substantially negating the risk of GVHD.

## Conclusion

With the identification of different ILC1 subsets in virtually every tissue in the body, it has become clear that these cells do not express a unique characteristic marker that singularly defines this subset. Some ILC1s, such as liver-resident ILCs, have a relatively well-defined phenotype, location, and origin, and this is clearly distinct from NK cells. In other tissues, such as the thymus or salivary glands, ILC1s appear to share a number of features with NK cells (Table 1, Figure 1). This includes the expression of Eomes and CD49b. Thus, whether all ILC1 subsets represent one distinct lineage with the same progenitor and blueprint for transcriptional regulation is not clear. It seems unlikely. Liver and salivary gland ILC1s do not require NFIL3 for their development while ILC1s in the gut it is essential (10, 34, 67). More intriguingly, ILC1s exhibit a differential need for IL-15 depending tissue they reside (31). Nevertheless, ILC1s appear to be very adaptable and their phenotype strongly reflects their varied microenvironments in which they exist. They appear to develop from precusors in the fetal liver, bone marrow, or thymus, but can also, somewhat surprisingly, transdifferentiate from other ILC lineages, including ILC2s and ILC3s. Thus, in order to understand the contribution of ILC1 to immune protection, further work will be required to better characterize the origin, transcriptional regulation and perhaps most importantly how microenvironmental factors drive their development and plasticity. This will depend significantly on the generation of novel models in which ILC1s can be specifically deleted without affecting the NK cell compartment and allow detailed understanding of the precise role of ILC1s during infections and inflammatory diseases.

## Author Contributions

All authors listed, have made substantial, direct and intellectual contribution to the work, and approved it for publication.

## Conflict of Interest Statement

The authors declare that the research was conducted in the absence of any commercial or financial relationships that could be construed as a potential conflict of interest.
